# Is lower birthweight truly causal for increased cardiovascular risk?

**DOI:** 10.1093/eurheartj/ehae509

**Published:** 2024-10-03

**Authors:** Nicole M Warrington, Geng Wang, Tom A Bond

**Affiliations:** Institute for Molecular Bioscience, University of Queensland, 306 Carmody Rd, St Lucia, Brisbane, QLD 4072, Australia; Frazer Institute, University of Queensland, 37 Kent St, Woolloongabba, Brisbane, QLD 4102, Australia; Institute for Molecular Bioscience, University of Queensland, 306 Carmody Rd, St Lucia, Brisbane, QLD 4072, Australia; Frazer Institute, University of Queensland, 37 Kent St, Woolloongabba, Brisbane, QLD 4102, Australia; MRC Integrative Epidemiology Unit at the University of Bristol, Bristol, UK; Population Health Sciences, Bristol Medical School, University of Bristol, Bristol, UK; Department of Epidemiology and Biostatistics, Imperial College London, London, UK

**This commentary refers to ‘Birth weight influences cardiac structure, function, and disease risk: evidence of a causal association’, by M. Ardissino *et al.*, https://doi.org/10.1093/eurheartj/ehad631 and the discussion piece ‘Genetic analyses of birthweight and cardiovascular disease’, by M. Ardissino *et al*., https://doi.org/10.1093/eurheartj/ehae510.**

Ardissino *et al*.^1^ present a Mendelian randomization (MR) study suggesting that lower birthweight is causal for increased risk of coronary artery disease. We argue that this association is unlikely to reflect a causal effect of birthweight on disease risk but is rather due to genetic variants in the offspring genome that influence both traits (i.e. genetic pleiotropy).

Low birthweight is unlikely to directly affect the risk of cardiovascular outcomes; rather, it is an imperfect marker for unfavourable developmental processes during pregnancy. However, if birthweight is truly causal for cardiovascular outcomes, then most factors that lower birthweight should also be associated with increased cardiovascular risk. Nevertheless, analyses in over 26 000 mother-offspring pairs, accounting for the correlation between maternal and offspring genotypes, found no associations between maternal genetic variants that lower offspring birthweight (potentially better proxies of an adverse intrauterine environment^2^) and offspring cardiovascular risk.^3^ This strongly argues against a causal effect of birthweight.^3^ Importantly, these studies also found associations between an individual’s own genetic variants and cardiovascular risk—similar to Ardissino *et al*.^1^—suggesting that genetic variants pleiotropically influence birthweight and cardiovascular outcomes.^3^

Second, if birthweight causally influences cardiovascular outcomes, then MR estimates of causal effects should be homogenous across individual genetic variants. Ardissino and colleagues did not report results of heterogeneity testing across the individual genetic variants, despite previous MR studies indicating significant heterogeneity.^4^ Whilst the authors use a multiplicative random effects model, this does not fully account for directional pleiotropy, and tests of the MR-Egger regression intercept are underpowered and make strong assumptions.

Finally, Ardissino and colleagues do not account for the possibility that maternal genotypes at the same/nearby correlated variants could influence offspring cardiovascular outcomes through mechanisms distinct from birthweight. Since maternal and offspring genotypes are correlated, it is important to control for maternal genotypes as a possible source of confounding. Merely restricting analyses to variants that have a foetal genetic effect on birthweight is not sufficient as these same variants may still exert maternal effects on offspring cardiovascular outcomes (NB. The authors have not detailed which genetic variants with ‘direct foetal genetic effects’ were included in their analyses, and some of these variants may also have maternal effects on birthweight, potentially confounding their analyses further). Using simulations, we have shown that disregarding maternal genotypes can induce spurious evidence for a causal effect of birthweight on cardiovascular outcomes.^5^ Therefore, the authors’ claim that using foetal genetic effects on birthweight shows that birthweight has a causal role ‘independent of the intrauterine environment’ is incorrect. Moreover, if true, this finding would lack practical relevance since interventions targeting birthweight would presumably involve manipulating the intrauterine environment.

As we highlight here and previously,^2,3^ extra caution is required when conducting MR analyses using birthweight as an exposure due to the correlation between offspring and maternal genotypes potentially reintroducing confounding into the analyses. We therefore believe that the authors’ results^1^ likely reflect the action of genetic pleiotropy through the offspring genome, rather than a causal effect of birthweight on risk of cardiovascular outcomes.
